# A reverse transcription loop-mediated isothermal amplification for broad coverage detection of Asian and African Zika virus lineages

**DOI:** 10.1186/s12879-020-05585-4

**Published:** 2020-12-11

**Authors:** Boon-Teong Teoh, Kim-Ling Chin, Nur-Izyan Samsudin, Shih-Keng Loong, Sing-Sin Sam, Kim-Kee Tan, Chee-Sieng Khor, Juraina Abd-Jamil, Nurhafiza Zainal, Annelies Wilder-Smith, Keivan Zandi, Sazaly AbuBakar

**Affiliations:** 1 Tropical Infectious Diseases Research and Education Centre (TIDREC), Universiti Malaya, Kuala Lumpur, Malaysia; 2 Institute for Advanced Studies (IAS), Universiti Malaya, Kuala Lumpur, Malaysia; 3 Department of Medical Microbiology, Faculty of Medicine, Universiti Malaya, Kuala Lumpur, Malaysia; 4 Department of Public Health and Clinical Medicine, Epidemiology and Global Health, Umeå University, Umeå, Sweden; 5 Lee Kong Chian School of Medicine, Nanyang Technological University, Singapore, Republic of Singapore; 6 Center for AIDS Research, Laboratory of Biochemical Pharmacology, Department of Pediatrics, Emory University School of Medicine, Atlanta, GA USA

**Keywords:** Infectious disease, Diagnostics, RT-LAMP, ZIKV, Mosquito, Vector, Vector-borne

## Abstract

**Background:**

Early detection of Zika virus (ZIKV) infection during the viremia and viruria facilitates proper patient management and mosquito control measurement to prevent disease spread. Therefore, a cost-effective nucleic acid detection method for the diagnosis of ZIKV infection, especially in resource-deficient settings, is highly required.

**Methods:**

In the present study, a single-tube reverse transcription loop-mediated isothermal amplification (RT-LAMP) assay was developed for the detection of both the Asian and African-lineage ZIKV. The detection limit, strain coverage and cross-reactivity of the ZIKV RT-LAMP assay was evaluated. The sensitivity and specificity of the RT-LAMP were also evaluated using a total of 24 simulated clinical samples. The ZIKV quantitative reverse transcription-polymerase chain reaction (qRT-PCR) assay was used as the reference assay.

**Results:**

The detection limit of the RT-LAMP assay was 3.73 ZIKV RNA copies (probit analysis, *P* ≤ 0.05). The RT-LAMP assay detected the ZIKV genomes of both the Asian and African lineages without cross-reacting with other arthropod-borne viruses. The sensitivity and specificity of the RT-LAMP assay were 90% (95% CI = 59.6–98.2) and 100% (95% CI = 78.5–100.0), respectively. The RT-LAMP assay detected ZIKV genome in 9 of 24 (37.5%) of the simulated clinical samples compared to 10 of 24 (41.7%) by qRT-PCR assay with a high level of concordance (κ = 0.913, *P* < 0.001).

**Conclusion:**

The RT-LAMP assay is applicable for the broad coverage detection of both the Asian and African ZIKV strains in resource-deficient settings.

**Supplementary Information:**

The online version contains supplementary material available at 10.1186/s12879-020-05585-4.

## Background

The *Aedes* mosquito-borne Zika virus (ZIKV) was discovered in Uganda in 1947 [1] and until recently, caused only sporadic human infections in Africa and Asia characterized by mild and self-limiting disease [2–4]. An epidemic affecting almost three quarters of the population in Yap Island, Federated States of Micronesia in 2007 was the first outbreak of ZIKV outside of Africa and Asia [5]. Following that, in 2013–2014, ZIKV rapidly spread to other countries in the Pacific, including French Polynesia [6], New Caledonia [7], the Cook Islands, Solomon Islands and Easter Island [8, 9]. Notably, the French Polynesia outbreak resulted in almost 9000 suspected cases, with an unusual increase of incidences involving neurological deformities among infants born after the outbreak [10]. In early 2015, ZIKV was identified in an outbreak in Bahia, Brazil [11] and by November 2016, transmission of ZIKV has since been reported in at least 48 other countries in the Americas [12]. It was during the Brazil outbreak that health authorities observed a surge in adverse pregnancy outcomes including congenital microcephaly, cerebral calcification and fetal growth restriction, among infants born to ZIKV-infected mothers [13]. Adults were clinically diagnosed with Guillain-Barré syndrome following ZIKV infection [8, 14], although the associated pathophysiological mechanisms have yet to be ascertained. The possible associations of ZIKV infection to these health disorders compelled the World Health Organization to declare ZIKV a Public Health Emergency of International Concern on February 2016 [15].

The ZIKV epidemics highlighted the unfortunate circumstances that this disease undeservedly affected most the economically marginalized populations [16] and by chance, they happened to be living in regions where *Aedes* mosquitoes thrive [17]. The expansion and spread of ZIKV, however, can be attributed to the wider geographical reach of *Ae. albopictus* that unlike *Ae. aegypti* can survive in temperate areas [17]. In addition, the infection can be transmitted through sexual contact [18, 19], mother-to-fetus [20–22] and via blood transfusion [23–25], albeit only in rare instances. Considering its far reaching impact of the infection, it is crucial that the development of rapid, cost-effective assays and portable ZIKV detection instruments to be expedited for deployment to the most needing.

Current ZIKV diagnosis use either serological assays detecting ZIKV-reactive antibodies or molecular detection of ZIKV RNA [26]. Serological assays detecting specific anti-ZIKV antibodies and the neutralization tests detecting specific ZIKV neutralizing antibodies are laborious, time-consuming and complicate by possible cross-reactivity with other flaviviruses [26, 27]. ZIKV RNA detection by quantitative reverse transcription-polymerase chain reaction (qRT-PCR) [28, 29] is often considered the gold standard as it provides definitive diagnosis. This method however, requires usage of costly reagents and amplification and detection equipment. The method also requires trained laboratory personnel to perform. The loop-mediated isothermal amplification (LAMP) method that provides simple, sensitive and rapid nucleic acid amplification under isothermal conditions promises to be a good alternative to qRT-PCR [30]. The method has been used for the detection of various RNA viruses including ZIKV [31–43]. Majority of the earlier reports of RT-LAMP for ZIKV used primers designed based on the lineage-specific regions of ZIKV genome [31–40]. In the present study, we described an improved RT-LAMP assay which took into account the detection of ZIKV strains from both the African and Asian lineages. The ZIKV RT-LAMP assay described here was evaluated for ZIKV RNA detection from simulated serum, saliva, and urine specimens.

## Methods

### Zika viruses

Three Asian ZIKV strains (P6–740, PRVABC59 and H/PF/2013) [44–46] and one African ZIKV strain (MR766) [1] were used. Strain P6–740 was obtained from Dr. Robert Tesh (World Reference Center for Emerging Viruses and Arboviruses, The University of Texas Medical Branch, Galveston, USA). Strain PRVABC59 was maintained in the laboratory by K. Zandi (author). Strain H/PF/2013 and MR766 were provided by Dr. Li-Sze Lim (Medical Innovation Ventures Pte. Ltd., Malaysia). The viruses were propagated in Vero cells using Dulbecco’s modified Eagle medium (DMEM, Gibco, NY, USA) supplemented with 2% heat-inactivated fetal bovine serum (FBS collected in South America, Capricorn, Germany), 0.1 mM non-essential amino acids (NEAA) and 2 mM L-glutamine. The infected cells were incubated at 37 °C in 5% CO_2_ atmosphere for 7 days and the infected cell culture supernatants were then harvested and stored at − 80 °C until further use.

### Simulated clinical samples

The study was approved by the UM Institutional Biosafety and Biosecurity Committee (Approval Number: UMIBBC/NOI/R/TNCPNI/TIDREC-007/22072020) and the UMMC Medical Ethics Committee (Ethics Committee/IRB Reference Number: 908.11). All ZIKV-positive simulated clinical samples were prepared by spiking the viral culture supernatant into actual human saliva, urine and serum. The viral culture supernatant was serially diluted with the serum-free media to viral titers ranging from 10^− 2^ to 10^4^ plaque-forming unit/ml (PFU/ml). The diluted viral culture supernatants were then mixed with the saliva, urine and serum samples at a 1:9 ratio. The final viral titers of the simulated clinical samples ranged from 10^− 3^ to 10^3^ PFU/ml. The human saliva, urine and serum without spiked ZIKV were used as ZIKV-negative simulated clinical samples. A total of 24 simulated clinical samples consisting 8 saliva, 8 urine and 8 serum samples were prepared (Additional file 1: Table S1). The saliva and serum samples were obtained from the same healthy donor, while the urine sample was obtained from another healthy donor. Only Asian ZIKV strains P6–740 was used for the preparation of simulated clinical samples.

### Viral plaque assay

The viral plaque assay was performed to determine the infectious titer of the ZIKV used for the preparation of simulated clinical samples. Briefly, the Vero cells were seeded at a density of 2 × 10^5^ cells/well in 24-wells plate and allowed to grow overnight until more than 80% confluency in DMEM supplemented with 10% FBS, 0.1 mM NEAA and 2 mM L-glutamine. The overnight culture media was replaced with a 10-fold serially diluted virus stock in the serum-free media (200 μl of each virus dilution). The plate was placed on a rocker and gently rocked for 1 h for virus adsorption. Subsequently, the virus supernatants were removed from each well and overlaid with 1 ml of DMEM supplemented with 2% FBS, 0.8% carboxymethylcellulose (CMC, Sigma-Aldrich, USA), 0.1 mM NEAA and 2 mM L-glutamine. The plate was incubated at 37 °C in 5% CO_2_ atmosphere for 5 days. After 5 days of incubation, the overlaid media was removed and cells were fixed with 4% paraformaldehyde (Sigma-Aldrich, USA) for 30 min at room temperature. The cells were then washed using 1 × PBS for three times. Finally, 0.5% crystal violet in 20% ethanol (Sigma-Aldrich, USA) was used to stain the cells for 15 min in order to enable the visibility of virus plaques. The virus plaques were counted using the SMZ 1000 stereomicroscope (Nikon, Tokyo, Japan) and the virus infectious titer was expressed in PFU/ml.

### RNA extraction

The infected cell culture supernatant and simulated clinical serum samples (140 μl) were subjected to RNA extraction using QIAamp Viral RNA Mini Kit (Qiagen, Germany) following the manufacturer’s protocol. The eluted RNA (60 μl) was stored at − 80 °C until further use.

### Design of ZIKV-specific RT-LAMP assay primers

The virus genomes of both Asian and African lineages were retrieved from GenBank. The genomes of other flaviviruses were also included for comparison. The sequences were aligned using Clustal X 2.0 [47]. The RT-LAMP primers were designed following the criteria previously described [30]. Conserved regions among flaviviruses were identified and excluded from the primer sequences. The RT-LAMP primers designed were exhaustically compared with an alignment of ZIKV genomes retrieved from GenBank. The five nucleotides at the 3′ end of F3, B3, FLP and BLP primers as well as the five nucleotides at both 3′ and 5′ end of FIP and BIP primers were considered critical sites for priming and amplification. The coverage of the primers was further validated by assessing the assay using both Asian and African ZIKV strains.

### RT-LAMP assay

The RT-LAMP reaction was prepared using the Loopamp RNA Amplification Kit (Eiken Chemical Co. Ltd., Japan). Each RT-LAMP reaction (25 μl) was added with the inner primers (20 pmol each), outer primers (2.5 pmol each), loop primer (20 pmol each), Fluorescent Detection Reagent (Eiken Chemical Co. Ltd., Japan; 1 μl), and the eluted RNA (5 μl). The RT-LAMP were performed using LA-500 Loopamp real-time turbidimeter (Eiken Chemical Co. Ltd., Japan) according to the following conditions: 90 min at 63 °C followed by 5 min of assay inactivation at 80 °C. The turbidity of RT-LAMP reaction was measured at 650 nm every 6 s. The threshold time (Tt) value was recorded when the turbidity crossed the threshold cut-off value at 0.07 absorbance units [48, 49].

### Cross-reactivity of RT-LAMP assay

The cross-reactivity of the ZIKV RT-LAMP primers was evaluated against other arboviruses including dengue virus type 1 (DENV-1), DENV-2, DENV-3, DENV-4, Japanese encephalitis virus (JEV), Langat virus (LGTV), Sindbis virus (SINV), Chikungunya virus (CHIKV), and Getah virus (GETV). All these viruses were attained from the TIDREC viral repository [48–52]. Nuclease-free water was used as the negative control.

### Detection limit of RT-LAMP assay

The detection limit of the RT-LAMP assay was assessed by using a 10-fold serially diluted ZIKV RNA (ranged from 1 to 10^3^ RNA copies) extracted from the virus culture supernatant. The initial ZIKV RNA copy number in the culture supernatant stock used were quantitated using the qRT-PCR assay. The detection limit test of RT-LAMP was repeated four times. Nuclease-free water was used as the negative control and as the diluent for the preparation of serially diluted viral RNA.

### Evaluation of RT-LAMP assay

The feasibility of the RT-LAMP assay for detection of ZIKV RNA in clinical setting was assessed using the simulated clinical samples as described above. The qRT-PCR was used as a reference assay for the detection of ZIKV RNA in the samples. Results from the RT-LAMP and qRT-PCR assays were compared.

### Real-time qRT-PCR assay (reference assay)

The viral RNA and samples used were quantitated using the Genesig Real-Time qRT-PCR ZIKV Detection Kit (PrimerDesign Ltd., UK). The Genesig qRT-PCR assay standard plot was prepared using a 10-fold serially diluted synthetic ZIKV RNA template with known copy number (ranged from 10 to 10^6^ RNA copies). The qRT-PCR reaction consists of the real time master mix (10 μl), probe/primer mix (1 μl), nuclease-free water (4 μ), and the extracted RNA (5 μl), in a 20 μl final reaction volume. The qRT-PCR were performed according to the following conditions: 10 min at 55 °C, 8 min at 95 °C followed by 50 cycles of 10 s at 95 °C and 60 s at 60 °C using StepOnePlus real time PCR system (Applied Biosystems, USA). The threshold cycles (Ct) obtained were used to determine the ZIKV RNA copy number in the samples based on standard curve from qRT-PCR using StepOne Software v2.2.1.

### Statistical analysis

A probit analysis (*P* ≤ 0.05) was performed to determine the detection limit of the RT-LAMP assay. The degree of agreement [kappa value (κ), *P* < 0.001] between RT-LAMP and qRT-PCR test results was measured. The degree of agreement and probit analyses were performed using IBM SPSS Statistics, version 21 (IBM Corporation, New York, United States). The diagnostic performance of RT-LAMP compared to qRT-PCR was determined using web-based EBM Diagnostic Test Calculator (http://ebm-tools.knowledgetranslation.net/calculator/diagnostic/).

## Results

### Design of ZIKV-specific RT-LAMP assay primers

**Fig. 1:**
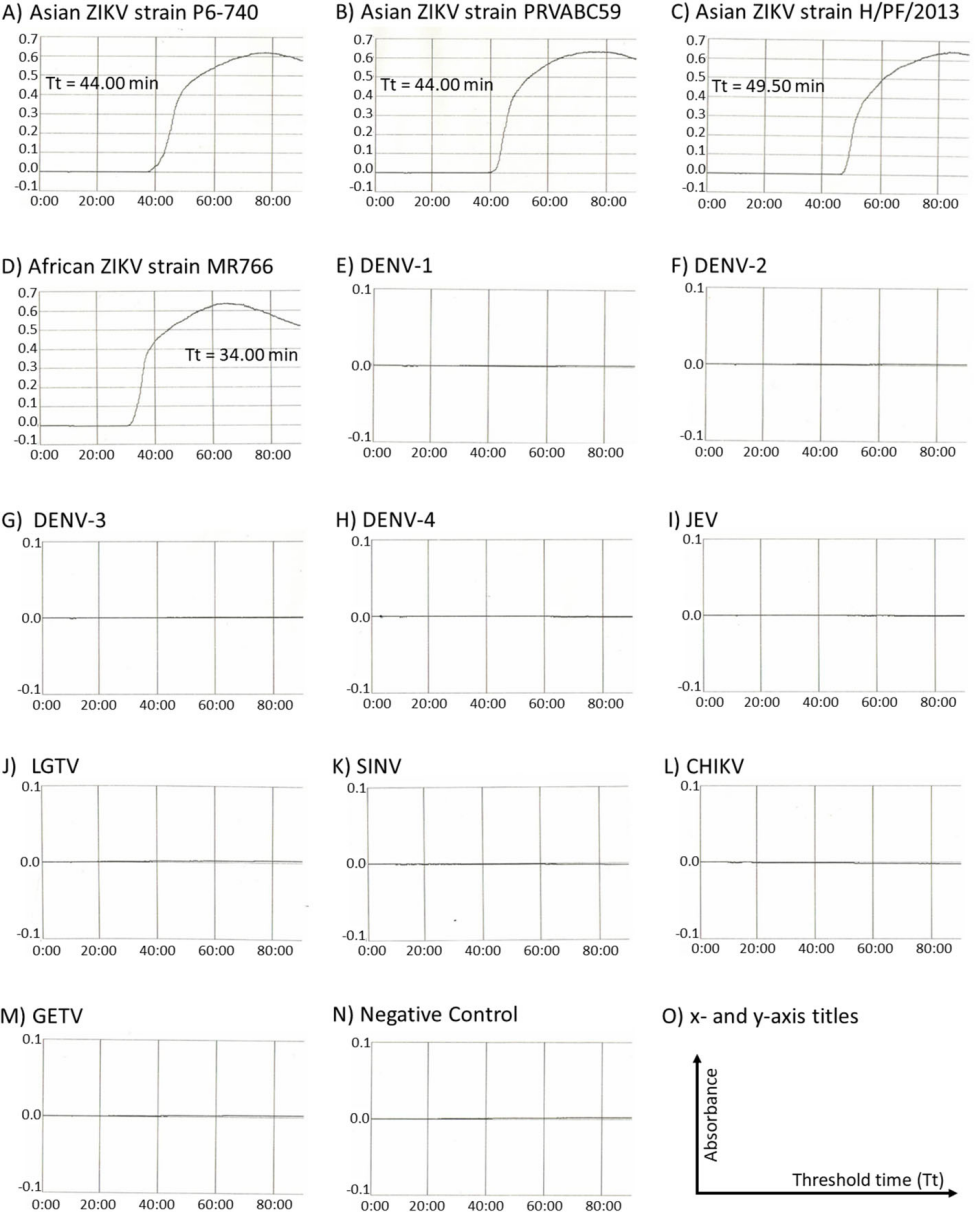
Amplification curves of the ZIKV RT-LAMP assay. A-D, four ZIKV strains; E-M, four DENV, JEV, LGTV, SINV, CHIKV and GETV, respectively; N, negative control; O, y- and x-axis titles

A set of six RT-LAMP primers comprising two outer, two inner, and two loop primers was designed (Table 1 and Additional file 2: Fig. S1) to target both Asian and African ZIKV strains. This was accomplished using the alignment of the conserved NS2A-NS3 junction of the ZIKV genome; each lineage-specific consensus was derived from at least five ZIKV strains (Additional file 3: Table S2). The sequences of the RT-LAMP primers were further compared with an alignment of 463 ZIKV genomes retrieved from GenBank (Additional file 4: Figure S2). In this comparison, BIP and FLP primers showed no critical nucleotide mismatch with all ZIKV genomes. Whereas the F3, B3, FIP and BLP primers respectively showed 0.22% (1/463), 1.94% (9/463), 1.30% (6/463) and 1.30% (6/463) of critical nucleotide mismatches with the ZIKV genomes. Figure 1 showed that the RT-LAMP primers detected the four reference ZIKV strains.

**Table 1:** RT-LAMP primers used for the rapid detection of ZIKV

Primer ^a^	Sequence (5′ → 3′)
F3	GGCRGAYATWGAGATGGCTGG
B3	CACTCCAACYTGTGTTGAACC
FIP	ACTT CC GC RT CYTT TT CC CATG TG AT GYTA YG TG GTCT CRGG AAAG AGTG T
BIP	GAGAGA GA TC ATAC TCAA RGTG GT CC TTCT TY AC ATAY AC RT ACCA CG CT CC
FLP	TGATRTCACCTGCTCTTTCAATGTACAT
BLP	TGTGGCATGAACCCAATAGC

^a^ F3, forward outer primer; B3, backward outer primer; *FIP* Forward inner primer, *BIP* Backward inner primer; *FLP* Forward loop primer, *BLP* Backward loop primer

### Cross-reactivity of RT-LAMP assay

No cross-reactivity of the RT-LAMP assay was observed with all other arthropod-borne viruses including all four DENV serotypes, JEV, LGTV, SINV, CHIKV and GETV (Fig. 1).

### Detection limit of RT-LAMP assay

**Fig. 2:**
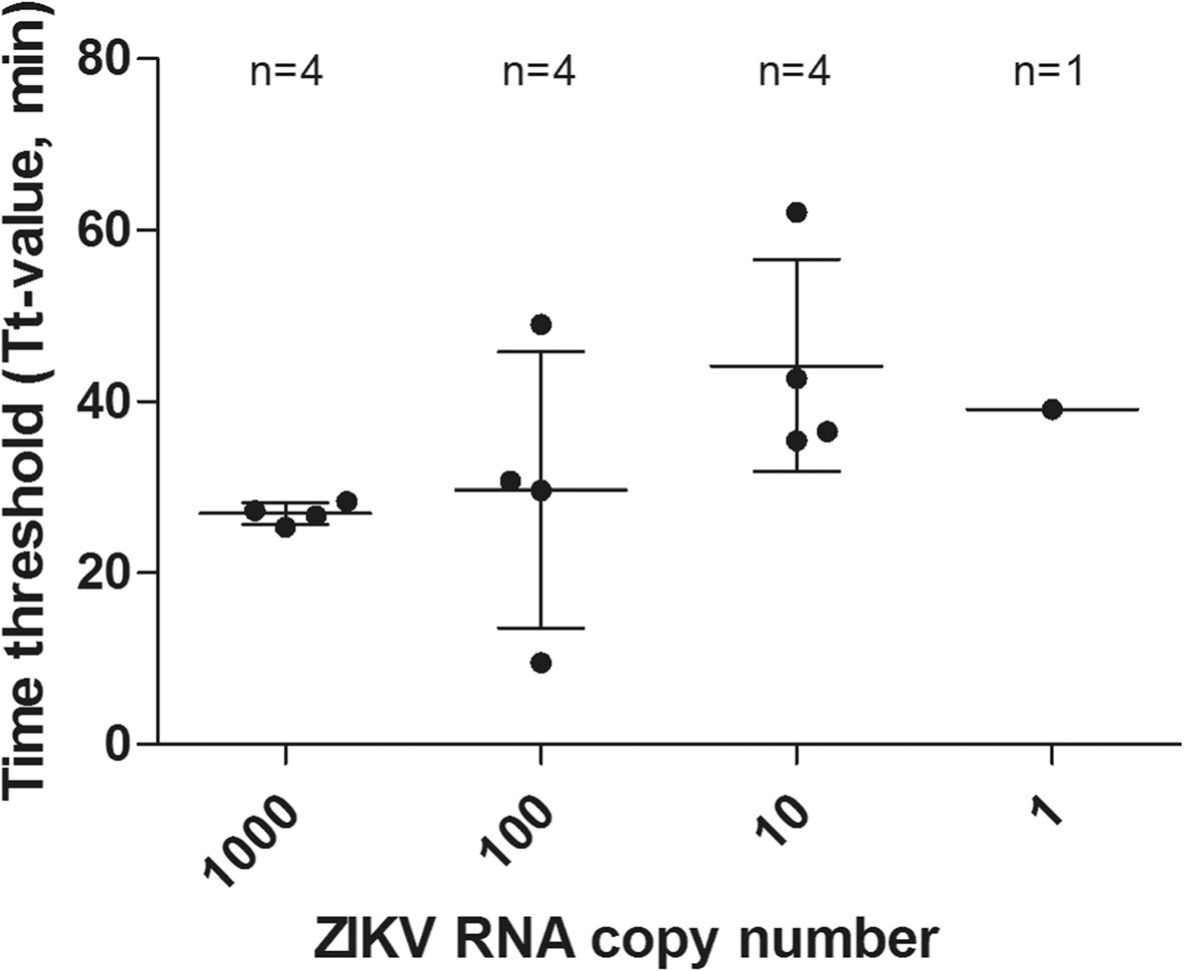
Time threshold of positivity for RT-LAMP assays of serially diluted ZIKV RNA. The mean of Tt-values was calculated with available positive results out of four replicates. Error bars indicate the standard deviations of Tt-values from the mean

**Fig. 3:**
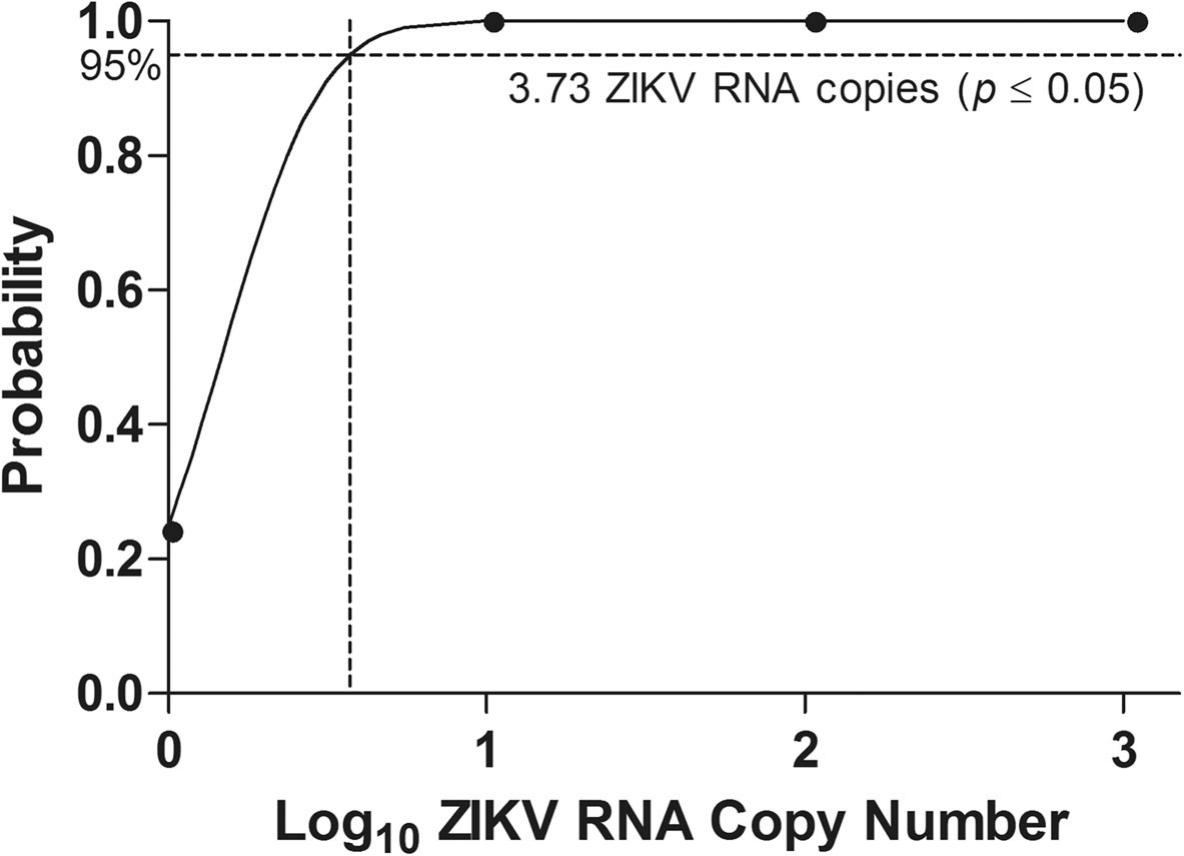
Detection limit of the ZIKV RT-LAMP assay. The probit regression curve was obtained from four replicates of ZIKV RNA in four dilutions (1000, 100, 10, and 1 copy numbers)

The detection limit of the RT-LAMP assay was assessed by repeatedly testing on the serially diluted ZIKV RNA with known copy number (Fig. 2 and Additional file 5: Table S3). The positive detection by RT-LAMP assays (*n* = 4) for 1000, 100, 10, and 1 ZIKV RNA copy were 100% (4 of 4), 100% (4 of 4), 100% (4 of 4), and 25% (1 of 4), respectively, with the mean time threshold (Tt) of 26.95 ± 1.25 min, 29.70 ± 16.14 min, 44.18 ± 12.37 min, 39.10 min, respectively. The detection limit of the RT-LAMP assay was 3.73 ZIKV RNA copies (probit analysis, *P* ≤ 0.05) (Fig. 3).

### Evaluation of RT-LAMP assay

**Fig. 4:**
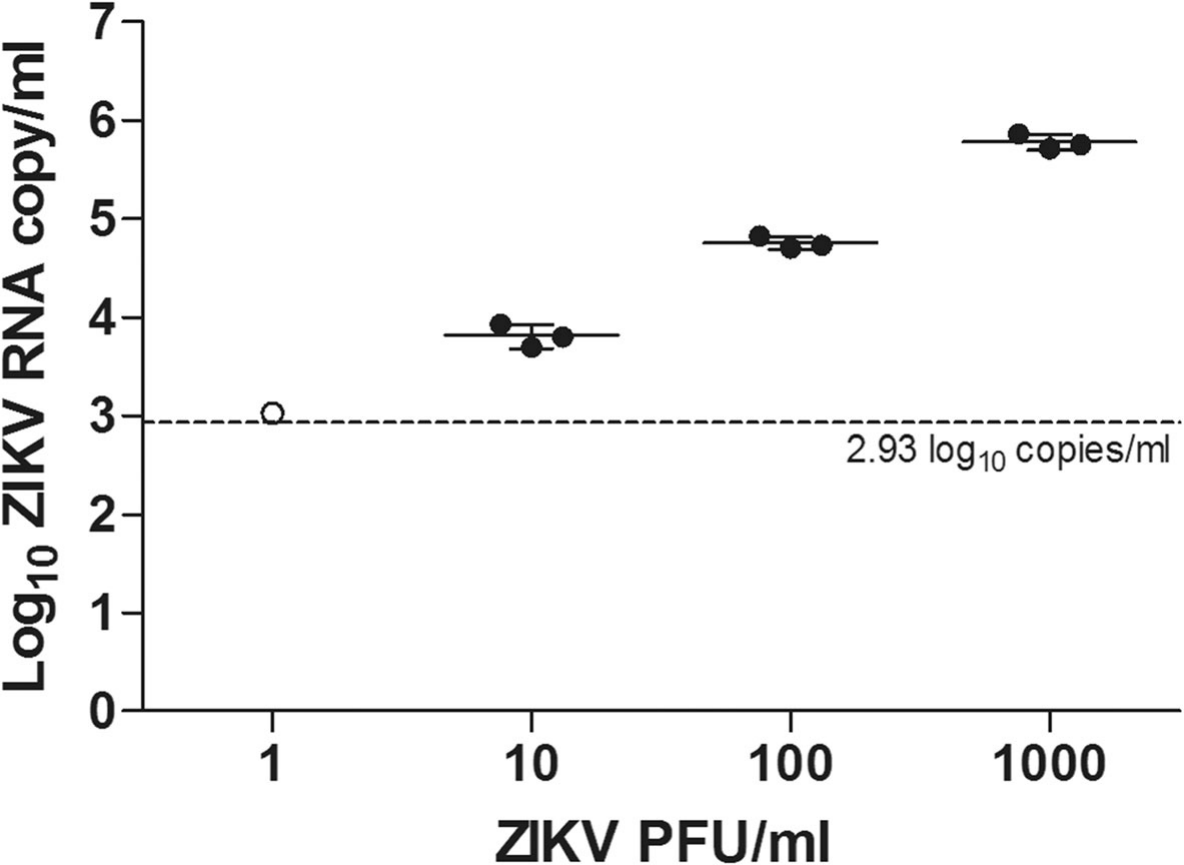
ZIKV RNA copy numbers of the simulated clinical human saliva, urine and serum specimens that tested positive by qRT-PCR according to the infectious virus titer (*n* = 10). The dashed line indicates the detection limit of qRT-PCR. The error bars indicate the standard deviation of the viral RNA copy numbers from the mean. Open circle, positive by qRT-PCR only; filled circle, positive by both qRT-PCR and RT-LAMP

The RT-LAMP assay for detection of ZIKV RNA was assessed by testing on a total of 24 simulated clinical samples. No evaluation was performed on actual clinical samples as none are currently available at the center. To date no Zika patient has been seen in our hospital. The diagnostic performance of the RT-LAMP in comparison with qRT-PCR assay was summarized in Table 2. The sensitivity and specificity of the RT-LAMP assay were 90% (95% CI = 59.6–98.2) and 100% (95% CI = 78.5–100.0), respectively. The RT-LAMP assay detected ZIKV genome in 9 of 24 (37.5%) of the samples compared to 10 of 24 (41.7%) by qRT-PCR assay, with a high concordance of *κ* = 0.913 (*P* < 0.001) between these two methods. ZIKV RNA was tested negative in one urine sample by RT-LAMP but positive by qRT-PCR; this sample contained 3.02 log_10_ copies of RNA/ml or 1 PFU/ml (Fig. 4). Samples containing 10–10^3^ PFU/ml (*n* = 9) were tested positive by both RT-LAMP and qRT-PCR, whereas samples containing 10^− 1^–10^− 3^ PFU/ml (*n* = 9) and the negative samples without spiked ZIKV (*n* = 3) were tested negative by both RT-LAMP and qRT-PCR.

**Table 2:** Diagnostic performance of the RT-LAMP assay against that of the qRT-PCR assay in the simulated clinical human saliva, urine and serum samples (*n* = 24)

		qRT-PCR		Sensitivity [% (95% CI^c^)]	Specificity [% (95% CI)]	PPV^d^ [% (95% CI)]	NPV ^e^ [% (95% CI)]
	Results^a, b^	Pos [n (%)]	Neg [n (%)]				
RT-LAMP	Pos	9 (37.5)	0 (0.0)	90.0 (59.6–98.2)	100.0 (78.5–100.0)	100.0 (70.1–100.0)	93.3 (70.2–98.8)
	Neg	1 (4.2)	14 (58.3)				

^a^ Agreement of results between RT-LAMP and qRT-PCR, *κ* = 0.913

^b^ Pos, positive; Neg, negative

^c^ CI, confidence interval

^d^ PPV, positive predictive value

^e^ NPV, negative predictive value

## Discussion

In the present study, a RT-LAMP assay was developed for isothermal detection of ZIKV RNA in simulated clinical specimens. The RT-LAMP assay could detect all the ZIKV strains used in this study without cross-reacting with a number of other common arthropod-borne viruses including all four DENV serotypes, JEV, LGTV, SINV, CHIKV and GETV. The RT-LAMP assay was sensitive, specific and useful for broad coverage detection of both the Asian and African ZIKV lineages.

Recently, several research groups have developed RT-LAMP assays for the detection of ZIKV by targeting the different regions of the ZIKV genome with excellent sensitivity and specificity [31–43]. They however, designed the primers for the RT-LAMP assays based only on a single lineage of ZIKV, which is either Asian [33–37, 39, 40] or African [32] lineage. Many studies designed the RT-LAMP primers based primarily on the sequences of the Asian ZIKV since the recent epidemics in the Pacific islands and Americas [5–7, 11] were caused by ZIKV from the Asian lineage [53]. Nonetheless, the possibility of re-emergence of the African lineage should not be excluded. Chotiwan et al. (2017) suggested using two sets of RT-LAMP primers, one for Asian ZIKV and another one for the African ZIKV [31]. The two-tube RT-LAMP assay, however, would definitely increase the cost of diagnosis. On the other hand, Kurosaki et al. (2017) combined two sets of RT-LAMP primers (total 11 primers) in single tube for simultaneous detection of Asian and African ZIKV [41]. Nonetheless, the use of more than six primers is often not preferable in RT-LAMP assay to avoid false positive due to a primer self-amplification [54]. Recently, Escalante-Maldonado et al. (2019) and Bui et al. (2020) developed RT-LAMP assay using a set of six primers based on the 64 and 130 ZIKV sequences retrieved from GenBank, respectively [42, 43]. Here, we designed a set of six RT-LAMP primers without critical mismatches with at least 95% of the 463 ZIKV genome sequences for broad coverage detection of both the Asian and African ZIKV lineages in a single-tube assay.

The detection limit of the RT-LAMP assay (~ 4 copies of ZIKV RNA) was comparable to that of ZIKV RT-LAMP assays previously reported; the detection limits ranged from 1 to 111 copies of ZIKV RNA [31, 32, 35]. In this study, the ZIKV RNA copy/infectious particle ratio in the simulated clinical specimens ranged from 10^2^ to 10^3^. Similar ZIKV RNA copy/infectious particle ratios have also been previously reported [55, 56]. Thus, the RT-LAMP assay is a potentially useful diagnostic tool for detecting viremic patients who are potentially contagious. Early detection of the viremic patients would permit immediate execution of proper disease control measures such as deploying insecticidal spraying and activating community-based mosquito control activities. Early diagnosis would also help clinicians in providing proper supportive treatment and counselling of Zika patients without needing to prescribe unnecessary medications.

Zika virus has been detected in human blood, saliva [57, 58], urine [59–61], semen [62, 63] and breastmilk [64]. Among all types of specimens, blood, saliva and urine are the common specimens used for Zika diagnosis. It has been reported that the viral titers in the patient’s blood, during the acute infection, ranged from 10^3^ to 10^6^ RNA copies/ml [65]. The viremic phase of ZIKV infection, however, is short with less than 2 to 5 days after fever onset [57, 59, 60]. The use of saliva has been shown to improve the detection of ZIKV RNA but did not enlarge the window of detection [57]. In contrast, shedding of ZIKV in urine persists for ~ 2 weeks [60] and sometimes it can be up to > 20 days after fever onset [59]. The use of urine specimen, therefore, is a practical way to enlarge the window of ZIKV detection. Here, we demonstrated that the RT-LAMP assay detected the simulated clinical specimens (serum, saliva, and urine) with viral load of as low as 10 PFU/ml, which is equivalent to approximately 10^3^ to 10^4^ RNA copies/ml. This detection sensitivity would be sufficient to diagnose viruric patients if their urine samples are collected within 10 days after fever onset considering viruric patients usually has viral load of > 10^3^ RNA copies/ml during the first 10 days of ZIKV infection [59].

There are, however, several limitations to our study. The performance of the RT-LAMP assay has not been validated on any real clinical samples. The evaluation was done in a very small cohort of simulated clinical samples (*n* = 24), in which the virus titers could be controlled. Therefore, there is no assurance of similar performance in field diagnostic settings.

## Conclusions

The RT-LAMP assay developed in this study was specific and sensitive for broad coverage detection of both the Asian and African ZIKV strains. The RT-LAMP assay can greatly enhance diagnosis of Zika in situation where resources are deficient.

## Supplementary Information

**Additional file 1:** **Table S1.** Preparation of the simulated clinical samples used in this study. A total of 24 simulated clinical samples consisting 8 saliva, 8 urine and 8 serum samples were prepared.

**Additional file 2:** **Figure S1.** Map of RT-LAMP primers in alignment with the ZIKV NS2-NS3 gene junction consensus sequences. The nucleotide positions refer to the published complete genome of ZIKV MR766 (GenBank accession number: NC_012532).

**Additional file 3:** **Table S2.** The ZIKV genomes used in this study.

**Additional file 4:** **Figure S2.** Nucleotide mismatches of the RT-LAMP primers against 463 ZIKV genome sequences. The top sequences are primer sequences. Total numbers of ZIKV genomes with 100% sequence similarity to the primers are shown at 3′’ end of the primers. Only the ZIKV genomes with nucleotide mismatches to the primer are listed. The dots indicate the same nucleotides as the top sequence. The boxes indicate the nucleotide positions which are critical for priming and amplification.

**Additional file 5:** **Table S3.** Time threshold of positivity for RT-LAMP assays of serially diluted ZIKV RNA.

## Data Availability

All data generated or analyzed in this study are included in this published article and its Additional files.

## Abbreviations

*Ae*.: *Aedes*
B3: Backward outer primer
BIP: Backward inner primer
BLP: Backward loop primer
CHIKV: Chikungunya virus
CI: Confidence interval
CMC: Carboxymethylcellulose
CO_2_: Carbon dioxide
DENV: Dengue virus
DMEM: Dulbecco’s modified Eagle medium
F3: Forward outer primer
FBS: Fetal bovine serum
FIP: Forward inner primer
FLP: Forward loop primer
GETV: Getah virus
IAS: Institute for Advanced Studies
JEV: Japanese encephalitis virus
LGTV: Langat virus
NEAA: Non-essential amino acids
NS: Non-structural
PBS: Phosphate-buffered saline
PFU: Plaque-forming unit
qRT-PCR: Quantitative reverse transcription-polymerase chain reaction
RNA: Ribonucleic acid
RT-LAMP: Reverse transcription loop-mediated isothermal amplification
SINV: Sindbis virus
TIDREC: Tropical Infectious Diseases Research and Education Centre
Tt: Time threshold
UM: Universiti Malaya
UMMC: Universiti Malaya Medical Centre
ZIKV: Zika virus
