# Supplementary material for: A reverse transcription loop-mediated isothermal amplification for broad coverage detection of Asian and African Zika virus lineages
Source: BMC Infect Dis. 2020 Dec 11;20:947. doi: 10.1186/s12879-020-05585-4 (PMC7731766; doi:10.1186/s12879-020-05585-4)
Supplement: Supplementary file 1 — Additional file 1: Table S1. Preparation of the simulated clinical samples used in this study. A total of 24 simulated clinical samples consisting 8 saliva, 8 urine and 8 serum samples were prepared. [file 12879_2020_5585_MOESM1_ESM.pdf]

A reverse transcription loop-mediated isothermal amplification for broad coverage detection of Asian and African Zika virus lineages

Boon-Teong Teoh[1,*], Kim-Ling Chin[1,2], Nur-Izyan Samsudin[1], Shih-Keng Loong[1], Sing-Sin Sam[1], Kim-Kee Tan[1], Chee-Sieng Khor[1], Juraina Abd-Jamil[1], Nurhafiza Zainal[3], Annelies Wilder-Smith[4,5], Keivan Zandi[3,6] and Sazaly AbuBakar[1,3,*]

[1] Tropical Infectious Diseases Research and Education Centre (TIDREC), Universiti Malaya, Kuala Lumpur, Malaysia

[2] Institute for Advanced Studies (IAS), Universiti Malaya, Kuala Lumpur, Malaysia

[3] Department of Medical Microbiology, Faculty of Medicine, Universiti Malaya, Kuala Lumpur, Malaysia

[4] Department of Public Health and Clinical Medicine, Epidemiology and Global Health, Umeå University, Umeå, Sweden

[5] Lee Kong Chian School of Medicine, Nanyang Technological University, Singapore

[6] Center for AIDS Research, Laboratory of Biochemical Pharmacology, Department of Pediatrics, Emory University School of Medicine, Atlanta, Georgia, USA

*Corresponding authors

Sazaly AbuBakar

Email: sazaly@um.edu.my

Boon-Teong Teoh, Ph.D

Tel: +603 7967 5754

Email: boonteong@um.edu.my

Table S1. Preparation of the simulated clinical samples used in this study. A total of 24 simulated clinical samples consisting 8 saliva, 8 urine and 8 serum samples were prepared.

Initial viral titres of the diluted viral supernatant (PFU/ml)	Volume of diluted viral supernatant used (µl)	Volume of each human saliva, urine and serum used (µl)	Final volume of the simulated clinical samples (µl)	Final viral titres of the simulated clinical samples (PFU/ml)
10^4	100	900	1000	10^3
10^3	100	900	1000	10^2
10^2	100	900	1000	10
10	100	900	1000	1
1	100	900	1000	10^{-1}
10^{-1}	100	900	1000	10^{-2}
10^{-2}	100	900	1000	10^{-3}
0	0	1000	1000	0
