# Supplementary material for: A reverse transcription loop-mediated isothermal amplification for broad coverage detection of Asian and African Zika virus lineages
Source: BMC Infect Dis. 2020 Dec 11;20:947. doi: 10.1186/s12879-020-05585-4 (PMC7731766; doi:10.1186/s12879-020-05585-4)
Supplement: Supplementary file 4 — Additional file 4: Figure S2. Nucleotide mismatches of the RT-LAMP primers against 463 ZIKV genome sequences. The top sequences are primer sequences. Total numbers of ZIKV genomes with 100% sequence similarity to the primers are shown at 3′’ end of the primers. Only the ZIKV genomes with nucleotide mismatches to the primer are listed. The dots indicate the same nucleotides as the top sequence. The boxes indicate the nucleotide positions which are critical for priming and amplification. [file 12879_2020_5585_MOESM4_ESM.pdf]

A reverse transcription loop-mediated isothermal amplification for broad coverage detection of Asian and African Zika virus lineages

Boon-Teong Teoh[1, *], Kim-Ling Chin[1, 2], Nur-Izyan Samsudin[1], Shih-Keng Loong[1], Sing-Sin Sam[1], Kim-Kee Tan[1], Chee-Sieng Khor[1], Juraina Abd-Jamil[1], Nurhafiza Zainal[3], Annelies Wilder-Smith[4, 5], Keivan Zandi[3, 6] and Sazaly AbuBakar[1, 3, *]

[1] Tropical Infectious Diseases Research and Education Centre (TIDREC), Universiti Malaya, Kuala Lumpur, Malaysia

[2] Institute for Advanced Studies (IAS), Universiti Malaya, Kuala Lumpur, Malaysia

[3] Department of Medical Microbiology, Faculty of Medicine, Universiti Malaya, Kuala Lumpur, Malaysia

[4] Department of Public Health and Clinical Medicine, Epidemiology and Global Health, Umeå University, Umeå, Sweden

[5] Lee Kong Chian School of Medicine, Nanyang Technological University, Singapore

[6] Center for AIDS Research, Laboratory of Biochemical Pharmacology, Department of Pediatrics, Emory University School of Medicine, Atlanta, Georgia, USA

*Corresponding authors

Sazaly AbuBakar

Email: sazaly@um.edu.my

Boon-Teong Teoh, Ph.D

Tel: +603 7967 5754

Email: boonteong@um.edu.my

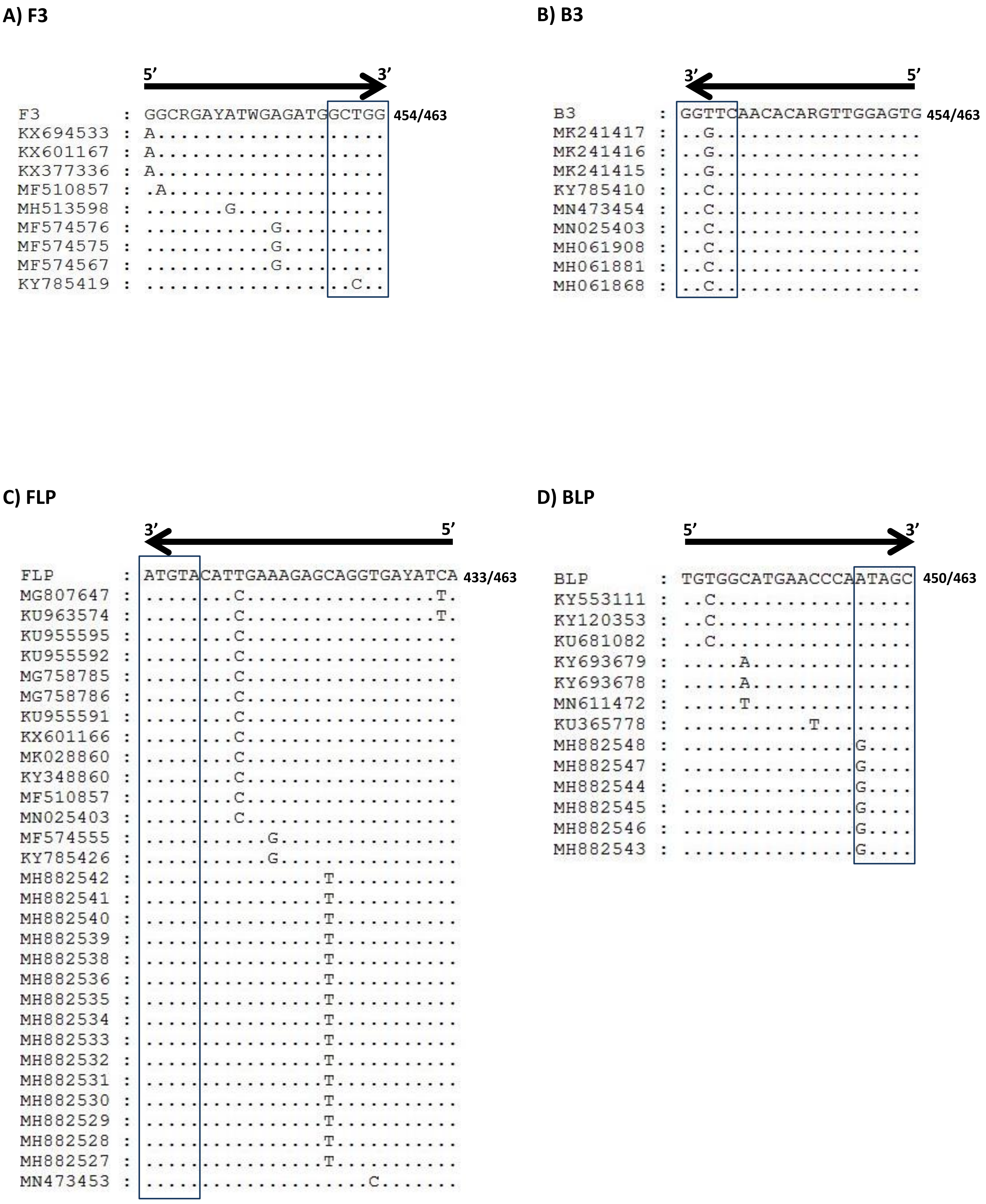
A) F3
5'
3'
F3 : GGCRGAYATWGAGATGGCTGG 454/463
KX694533 : A....................
KX601167 : A....................
KX377336 : A....................
MF510857 : .A...................
MH513598 : ....G.............
MF574576 : ....G.........
MF574575 : ....G.........
MF574567 : ....G.........
KY785419 : ....C..
B) B3
3'
5'
B3 : GGTTCAACACARGTTGGAGTG 454/463
MK241417 : ..G..................
MK241416 : ..G..................
MK241415 : ..G..................
KY785410 : ..C..................
MN473454 : ..C..................
MN025403 : ..C..................
MH061908 : ..C..................
MH061881 : ..C..................
MH061868 : ..C..................
C) FLP
3'
5'
FLP : ATGTACATTGAAAGAGCAGGTGAYATCA 433/463
MG807647 : ........C.................T.
KU963574 : ........C.................T.
KU955595 : ....C...................
KU955592 : ....C...................
MG758785 : ....C...................
MG758786 : ....C...................
KU955591 : ....C...................
KX601166 : ....C...................
MK028860 : ....C...................
KY348860 : ....C...................
MF510857 : ....C...................
MN025403 : ....C...................
MF574555 : ....G................
KY785426 : ....G................
MH882542 : ....T...........
MH882541 : ....T...........
MH882540 : ....T...........
MH882539 : ....T...........
MH882538 : ....T...........
MH882536 : ....T...........
MH882535 : ....T...........
MH882534 : ....T...........
MH882533 : ....T...........
MH882532 : ....T...........
MH882531 : ....T...........
MH882530 : ....T...........
MH882529 : ....T...........
MH882528 : ....T...........
MH882527 : ....T...........
MN473453 : ....C.......
D) BLP
5'
3'
BLP : TGTGGCATGAACCCAATAGC 450/463
KY553111 : ..C.................
KY120353 : ..C.................
KU681082 : ..C.................
KY693679 : .....A..............
KY693678 : .....A..............
MN611472 : .....T..............
KU365778 : ....T........
MH882548 : ....G....
MH882547 : ....G....
MH882544 : ....G....
MH882545 : ....G....
MH882546 : ....G....
MH882543 : ....G....

E) FIP

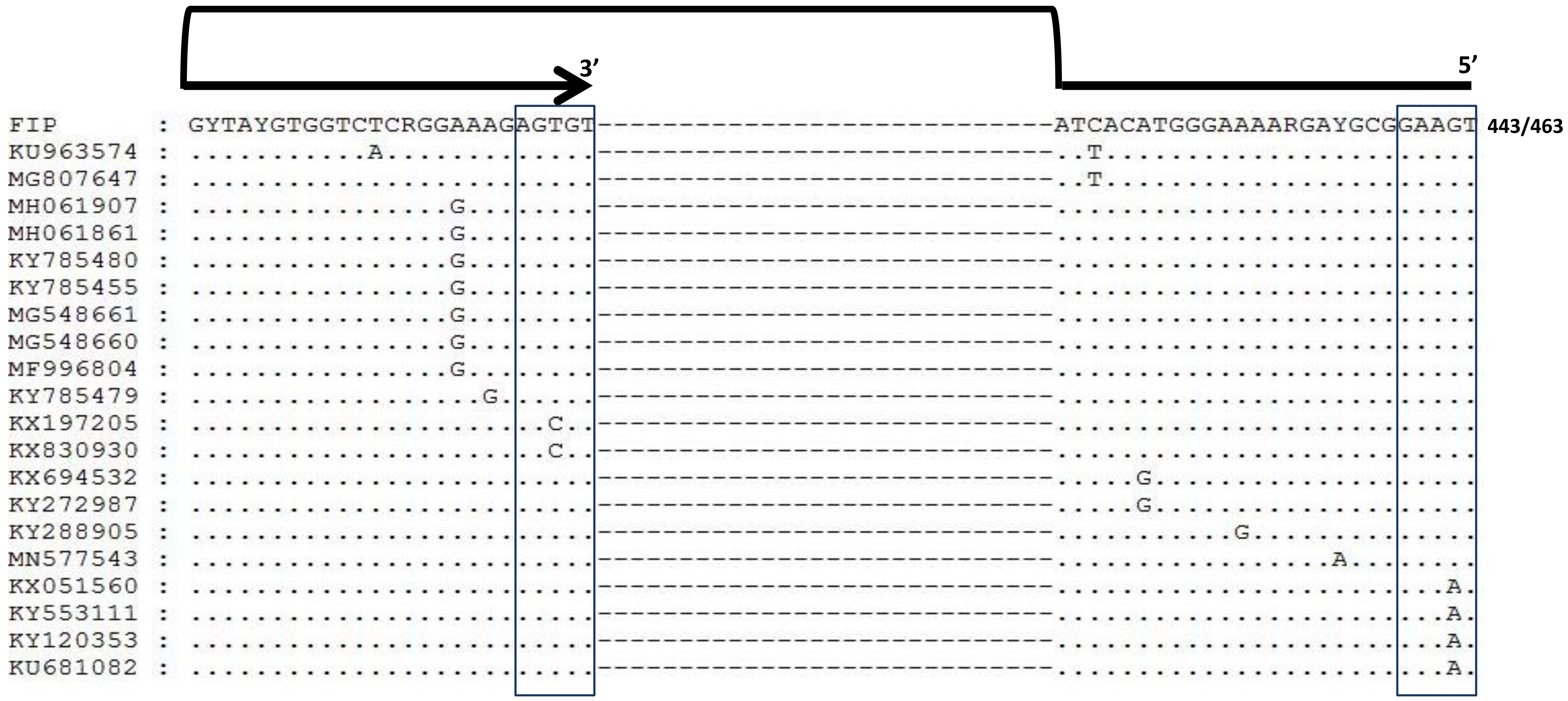
3’
5’
FIP : GYTAYGTGGTCTCRGGAAAGAGTGT ATCACATGGGAAAARGAYGCGGAAGT
443/463
KU963574
MG807647
MH061907
MH061861
KY785480
KY785455
MG548661
MG548660
MF996804
KY785479
KX197205
KX830930
KX694532
KY272987
KY288905
MN577543
KX051560
KY553111
KY120353
KU681082

F) BIP

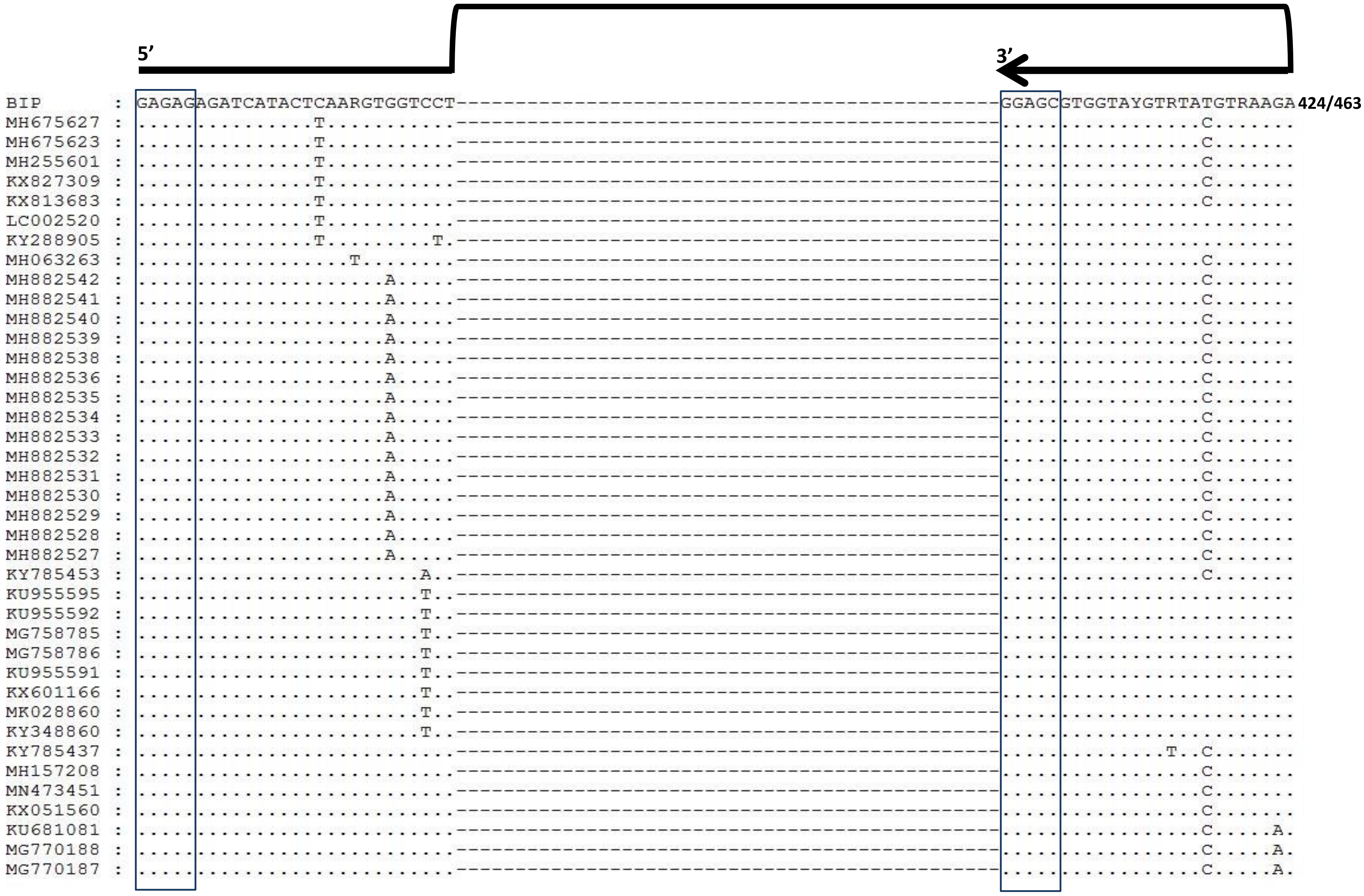
5’
3’
BIP : GAGAGAGATCATACTCAARGTGGTCCT GGAGCGTGGTAYGTRTATGTRAAGA
424/463
MH675627
MH675623
MH255601
KX827309
KX813683
LC002520
KY288905
MH063263
MH882542
MH882541
MH882540
MH882539
MH882538
MH882536
MH882535
MH882534
MH882533
MH882532
MH882531
MH882530
MH882529
MH882528
MH882527
KY785453
KU955595
KU955592
MG758785
MG758786
KU955591
KX601166
MK028860
KY348860
KY785437
MH157208
MN473451
KX051560
KU681081
MG770188
MG770187

Figure S2. Nucleotide mismatches of the RT-LAMP primers against 463 ZIKV genome sequences. The top sequences are primer sequences. Total numbers of ZIKV genomes with 100% sequence similarity to the primers are shown at 3’ end of the primers. Only the ZIKV genomes with nucleotide mismatches to the primer are listed. The dots indicate the same nucleotides as the top sequence. The boxes indicate the nucleotide positions which are critical for priming and amplification.
