# Supplementary material for: A reverse transcription loop-mediated isothermal amplification for broad coverage detection of Asian and African Zika virus lineages
Source: BMC Infect Dis. 2020 Dec 11;20:947. doi: 10.1186/s12879-020-05585-4 (PMC7731766; doi:10.1186/s12879-020-05585-4)
Supplement: Supplementary file 5 — Additional file 5: Table S3. Time threshold of positivity for RT-LAMP assays of serially diluted ZIKV RNA. [file 12879_2020_5585_MOESM5_ESM.pdf]

A reverse transcription loop-mediated isothermal amplification for broad coverage detection of Asian and African Zika virus lineages

Boon-Teong Teoh[1, *], Kim-Ling Chin[1, 2], Nur-Izyan Samsudin[1], Shih-Keng Loong[1], Sing-Sin Sam[1], Kim-Kee Tan[1], Chee-Sieng Khor[1], Juraina Abd-Jamil[1], Nurhafiza Zainal[3], Annelies Wilder-Smith[4, 5], Keivan Zandi[3, 6] and Sazaly AbuBakar[1, 3, *]

[1] Tropical Infectious Diseases Research and Education Centre (TIDREC), Universiti Malaya, Kuala Lumpur, Malaysia

[2] Institute for Advanced Studies (IAS), Universiti Malaya, Kuala Lumpur, Malaysia

[3] Department of Medical Microbiology, Faculty of Medicine, Universiti Malaya, Kuala Lumpur, Malaysia

[4] Department of Public Health and Clinical Medicine, Epidemiology and Global Health, Umeå University, Umeå, Sweden

[5] Lee Kong Chian School of Medicine, Nanyang Technological University, Singapore

[6] Center for AIDS Research, Laboratory of Biochemical Pharmacology, Department of Pediatrics, Emory University School of Medicine, Atlanta, Georgia, USA

*Corresponding authors

Sazaly AbuBakar

Email: sazaly@um.edu.my

Boon-Teong Teoh, Ph.D

Tel: +603 7967 5754

Email: boonteong@um.edu.my

Table S3. Time threshold of positivity for RT-LAMP assays of serially diluted ZIKV RNA.

	Time Threshold (Tt-value, min)			
ZIKV RNA copy number<br>Replicates	1000	100	10	1
1	28.24	49.00	35.24	-
2	25.24	30.42	42.42	-
3	26.42	29.36	62.06	39.06
4	27.18	39.30	36.30	-
